# The Protective Role of *Scorias spongiosa* Polysaccharide-Based Microcapsules on Intestinal Barrier Integrity in DSS-Induced Colitis in Mice

**DOI:** 10.3390/foods12030669

**Published:** 2023-02-03

**Authors:** Yingyin Xu, Huiyu Feng, Zhiyuan Zhang, Qian Zhang, Jie Tang, Jie Zhou, Yong Wang, Weihong Peng

**Affiliations:** 1 Sichuan Institute of Edible Fungi, Chengdu 610066, China; 2 National-Local Joint Engineering Laboratory of Breeding and Cultivation of Edible and Medicinal Fungi, Chengdu 610066, China; 3 Scientific Observing and Experimental Station of Agro-Microbial Resource and Utilization in Southwest China, Ministry of Agriculture, Chengdu 610066, China; 4 College of Food and Biological Engineering, Chengdu University, Chengdu 610106, China

**Keywords:** *S. spongiosa* polysaccharides, microcapsules, ulcerative colitis, intestinal microbiome, tight junction, NF-κB–STAT1–MAPK

## Abstract

*Scorias spongiosa*, a type of edible fungus, is beneficial for intestinal health. However, the mechanisms by which polysaccharides derived from *S*. *spongiosa* contribute to the integrity of the intestinal barrier have been little investigated. In the present study, 40 C57BL/6J mice were assigned into five groups: (1) Normal; (2) Dextran sulfate sodium (DSS)Administration; (3) DSS + Uncapped polysaccharides; (4) DSS + Low microcapsules; (5) DSS + High microcapsules. After one week of administration of *S. spongiosa* polysaccharides, all mice, excluding the Normal group, had free access to the drinking water of 3.5% DSS for seven days. Serum and feces were then taken for analysis. Scanning electron microscopy analysis indicated the structure of the micro-capped polysaccharides with curcumin was completed with a rough surface, which differs from the uncapped polysaccharides. Noticeably, *S. spongiosa* polysaccharides enhanced intestinal barrier integrity as evidenced by increasing the protein levels of Claudin-1, ZO-1 and ZO-2. Low-capped polysaccharides mitigated the DSS-induced oxidative stress by increasing catalase (CAT) concentration and decreasing malondialdehyde (MDA) and myeloperoxidase (MPO) concentrations. Besides, DSS treatment caused a disturbance of inflammation and the contents of IL-1β, IL-6, TNF-α and CRP were downregulated and the contents of IL-4, IL-10 and IFN-γ were upregulated by *S. spongiosa* polysaccharides. Research on the potential mechanisms indicated that *S. spongiosa* polysaccharides inhibited the DSS-triggered activation of NF-κB signaling. Moreover, the JAK/STAT1 and MAPK pathways were suppressed by *S. spongiosa* polysaccharides in DSS-challenged mice, with Lcap showing the strongest efficacy. 16S rDNA amplicon sequencing revealed that the richness and diversity of the microbial community were reshaped by *S. spongiosa* polysaccharide ingestion. Therefore, our study substantiated that *S. spongiosa* polysaccharides exhibited protective effects against colitis mice by reshaping the intestinal microbiome and maintaining the balance of intestinal barrier integrity, antioxidant capacity and colonic inflammation through regulation of the NF-κB–STAT1–MAPK axis.

## 1. Introduction

Ulcerative colitis (UC), one of the two main forms of inflammatory bowel disease (IBD), is a chronic immune-mediated idiopathic intestinal inflammatory disease, predominantly in the rectum and sigmoid colon, which tends to appear in clinical signs as diarrhea and abdominal pain [1,2]. The intestinal barrier defect is a major driver of UC development, in which colonic goblet cells and the permeable mucus barrier are depleted in patients with active UC [3,4,5]. In addition, studies have proved that dysbacteriosis in UC patients with decreased *Firmicutes* and increased *Gamma-proteobacteria* and *Enterobacteriaceae* promotes the imbalance of host immunity [6,7]. In summary, the pathogenesis of UC is the consequence of combined factors, including impairment of the gut epithelial barrier, changes in intestinal flora diversity and metabolic profiles and environmental factors [8]. Therefore, it is meaningful to build animal models similar to human UC to elucidate pathological mechanisms and explore clinical treatments. Dextran sulfate sodium (DSS) can cause colitis symptoms by inhibiting epithelial cell proliferation, destroying the intestinal mucosal barrier and intestinal flora homeostasis [9,10,11]. Experimental animals have weight loss, diarrhea and hematochezia, accompanied by colon shortening, barrier dysfunction, inflammation and immune cell infiltration, which are most similar to clinical symptoms and pathological changes of human UC [12,13,14].

Currently, most drugs function primarily as anti-inflammatory agents with no significant effect on the maintenance of intestinal barrier integrity and intestinal microbiome balance [15,16]. Therefore, the search for new therapeutic agents that not only alleviate inflammatory response but also maintain the intestinal barrier and gut microbiota balance can be considered as a goal of current exploration. In recent years, *Scorias spongiosa*, a type of edible fungus, attracted more and more attention from scientific researchers due to its medicinal and economic value [17]. It has been found that *S. spongiosa* contains many bioactive substances, such as polysaccharides, ergosterol, amino acids, vitamins and so on [18]. Extracts from *Scorias spongiosa* by various stimulating agents possess antioxidant, anticancer, antibacterial and immunomodulatory activities [19,20]. Studies indicated that *S. spongiosa* polysaccharides confer antioxidant and anti-inflammatory capacities and alter intestinal microbiota to benefit gut health [20,21].

However, little research investigated the effect of *S. spongiosa* polysaccharides on colitis and gut microbiome in DSS-challenged mice. Microencapsulation technology uses natural or synthetic polymer materials to embed solid, liquid or gas and other core substances [22]. Due to its many advantages including protection of the core material, enhanced stability and increased utilization rate, microcapsule technology has been widely used in pharmaceuticals, pesticides, food, cosmetics and other industries [23]. In recent years, it has been found that curcumin has a protective effect on the integrity of the intestinal barrier [24]. Curcumin is a fat-soluble component with a solubility of only 0.6 μg/mL in water [25]. Despite its many biological activities, curcumin’s low stability greatly limits its applications in humans [26]. After ingestion, only a small amount can be digested and absorbed, and the metabolic processes in the body are so rapid that it is soon excreted from the body [27]. It has difficulty exerting its physiological effects, resulting in very low bioavailability. Therefore, it is of interest to explore the formulation and preparation of curcumin microcapsules that have good embedding properties and can be used in the treatment of colitis.

In this study, polysaccharides were extracted from *S. spongiosa* and coated into microcapsules with curcumin to evaluate the mechanisms by which *S. spongiosa* polysaccharides protect the integrity of the intestinal barrier in DSS-challenged mice.

## 2. Materials and Methods

### 2.1. The Characterization of Scorias Spongiosa Polysaccharides

The *Scorias Spongiosa* was cleaned, dried at 60 °C to constant weight, and then crushed and screened at 60 mesh. An amount of 5 g of *Scorias Spongiosa* powder was put in 200 mL steam distillation water at 70 °C for 5 h, and ultrasonic for 30 min. The ultrasonic power was 500 W. After centrifugation at room temperature (3000 r/min, 10 min), the supernatant was collected and mixed with anhydrous ethanol to make the final concentration reach 80%. After standing at 4 °C for 12 h overnight, precipitation was taken followed by centrifugation (3000 r/min, 10 min) and dried at 60 °C to obtain the crude polysaccharide of *Scorias Spongiosa*. The extraction rate of fresh polysaccharides was 0.20%. The purity of polysaccharides was 92.16% by phenol-sulphuric acid assay. Subsequently, the monosaccharides in polysaccharides were analyzed by high-performance liquid chromatography (HPLC). *Scorias Spongiosa* polysaccharide mainly consists of three monosaccharides, including glucose, mannose and galactose.

### 2.2. The Formulation of Scorias Spongiosa Polysaccharide-Based Microcapsules

*S. spongiosa* polysaccharides were mixed with a certain quality of sodium alginate in the ratio of 3:50, dissolved with distilled water and kept at a constant temperature of 50 °C for 4 h. Then, curcumin and Tween 80 were added and mixed well under ultrasonic conditions. The mixture was homogenized in a high-pressure homogenizer at a pressure of 330 bar for 5 min at 4 °C.

The mixed emulsion was prepared by aspiration with a syringe using a Buchi microencapsulation granulator (B-395 Pro, Buchi, Flawil, Switzerland) and slowly dropped into the coagulation solution (CaCl_2_ solution) to form microcapsules. The volume of the coagulation solution was three times the volume of the mixed emulsion. The parameters were set to 200 μm nozzle aperture, 7 mL/min flow rate, 1200 Hz frequency, 700 mV voltage and 30% stirring speed. The microcapsules were cured at room temperature with stirring for a certain time, separated from the microcapsules, washed with water to wipe out the superficial CaCl_2_ on the microcapsules, and dehydrated in a vacuum freezing dryer at −50 °C and 0.5 mbar to obtain the microcapsule products.

### 2.3. Animals, Treatment and Diet

A total of 40 male C57BL/6J mice (aged 6~7 weeks, weighing 21~25 g) acquired from Chengdu Dashuo Laboratory Animal, were randomly allocated into five treatment groups with eight pens per group (one mouse per pen): (1) Normal group (Norm, normal diet); (2) DSS group (normal diet); (3) Uncapsule group (Uncap, normal diet + emulsion containing 1 mg/mL curcumin and 1 mg/mL *S. spongiosa* polysaccharides); (4) Low microcapsule group (Lcap, normal diet + low microcapsules containing 1 mg/mL curcumin and 1 mg/mL *S. spongiosa* polysaccharides); (5) High microcapsule group (Hcap, normal diet + high microcapsules containing 2 mg/mL curcumin and 1.5 mg/mL *S. spongiosa* polysaccharides). After 7 days of treatment with *S. spongiosa* polysaccharides, 3.5% (*w*/*v*) DSS in drink water was orally applied to all mice except the Norm group. Besides, animals were caged in an environment at constant temperature (24  ±  3 °C), humidity (54  ±  3%) and illumination (12 h light/dark cycle) and experimental diets and water were available ad libitum during the entire experimental period.

Before samples of feces and blood were collected, mouse anesthetization with pentobarbital sodium (30 mg/kg) was carried out at the end of the experiment and, afterwards, cervical dislocation was performed. Colon samples were snap-frozen into liquid nitrogen and stored at −80 °C for further analysis.

### 2.4. Determination of Serum Cytokines

Serum contents of catalase (CAT), malondialdehyde (MDA), myeloperoxidase (MPO), interleukin-1β (IL-1β), IL-4, IL-6, IL-10, tumor necrosis factor-α (TNF-α), interferon-γ (IFN-γ), diamine oxidase (DAO), trefoil factor 3 (TFF3) and C-reactive protein (CRP) were measured using spectrophotometric kits according to the manufacturer’s instructions (Jiangsu Meimian Industrial Co., Nanjing, China).

### 2.5. Western Blotting Analysis

The total proteins from the colon were lysed by radio immunoprecipitation assa (RIPA) lysis buffer and measured using the bicinchoninic acid (BCA) method to detect the protein concentration. The protein sample (40 μg) was mixed with 5 × loading buffer and separated in 10% SDS-PAGE gel. Then, the proteins were transferred onto the polyvinylidene difluoride (PVDF) membrane, which was subsequently blocked in 5% skim milk and incubated overnight at 4 °C in an appropriate dilution ratio of primary antibody. The abundances of Occludin (Thermo Fisher Scientific, Waltham, MA, USA), Claudin-1 (Thermo Fisher Scientific), Zonula occludens 1 (ZO-1; Thermo Fisher Scientific), ZO-2 (Thermo Fisher Scientific), phospho-Nuclear factor-κB (p-NF-κB; Abcam, Cambridge, UK), NF-κB (Abcam), phospho-IκBα (p-IκBα; CST, Boston, MA, USA), IκBα (CST), phospho-p38 Mitogen-activated protein kinase (p-p38 MAPK; CST), p38 MAPK (CST), phospho-Extracellular signal-regulated kinase (p-ERK; CST), Extracellular signal-regulated kinase (ERK; CST), phospho-Jun kinase (JNK; Proteintech, Wuhan, China), Jun kinase (JNK; Proteintech), phospho-Janus kinase (p-JAK; Proteintech), JAK (Proteintech), phospho-Signal transducer and activator of transcription-1 (p-STAT1; CST), STAT1 (CST) and actin beta (β-actin, Proteintech) proteins were assessed using an ECL kit and ChemiDoc Imaging Systems. To ensure the loading of equal protein samples, the expression level of β-actin was evaluated.

### 2.6. Scanning Electron Microscopy (SEM) Analysis

Scanning electron microscopy was used to observe the morphology of the uncapped *S. spongiosa* polysaccharides and polysaccharide-based microcapsules. Before observation, microcapsules were sputtered with gold for 30 s and, subsequently, observed in electron microscopy. The observing conditions were set at 20 kV and the magnification was 300.

### 2.7. 16S rDNA Analysis

The total genomic DNA in a bowel stool was extracted by the cetyltrimethylammonium bromide (CTAB)/sodium dodecyl sulfate (SDS) method. DNA concentration and purity were monitored on 1% agarose gel. 16S rRNA genes were amplified with specific primer (515F-806R). All PCR mixtures contained 15 μL of Phusion^®^ High-Fidelity PCR Master Mix (New England Biolabs, Ipswich, UK), 0.2 μM of each primer and 10 ng target DNA, and cycling conditions consisted of a first denaturation step at 98 °C for 1 min, followed by 30 cycles at 98 °C (10 s), 50 °C (30 s) and 72 °C (30 s), and a final 5 min extension at 72 °C. Then, the PCR products were quantified and qualified to generate sequencing libraries.

The paired-end reads were merged and quality filtered to obtain effective tags. To analyze the diversity and richness, alpha and beta diversity were calculated in QIIME2 software. Species annotation was performed using Silva Database [28].

### 2.8. Statistical Analysis

All results were assessed by one-way ANOVA followed by Tukey’s multiple comparison test using IBM SPSS Statistics version 20.0 (IBM, Armonk, NY, USA). All results were measured by means ± standard deviations (SD), and *p* < 0.05 indicates a significant difference between groups.

## 3. Results

### 3.1. S. spongiosa Polysaccharides Attenuated Weight Loss in Colitis Mice

**Figure 1:**
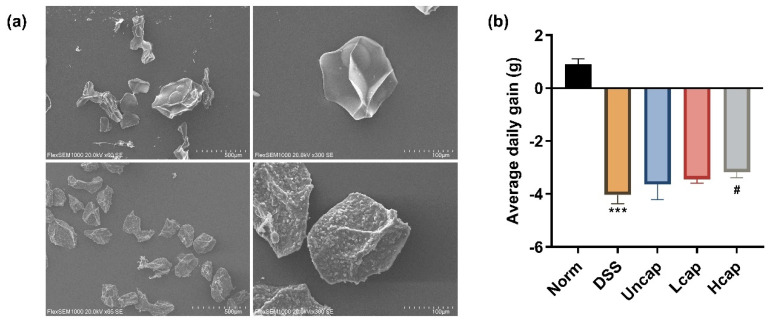
*S. spongiosa* polysaccharides attenuated weight loss in colitis mice. (**a**) Morphological characterization of *S. spongiosa* polysaccharides by scanning electron microscopy (SEM). (**b**) The average daily gain of mice during colitis. The values are reported as means ± SD (*n* = 8). *** *p* < 0.001 vs. Norm group; ^#^ *p* < 0.05 vs. DSS group.

First, we verified the efficiency of the capping process of *S. spongiosa* polysaccharides. The prepared microcapsules have a fully spherical structure, an uneven surface, and are fully granular. The uncapped polysaccharides have a compact structure with a flat surface, in contrast to the curcumin-coated microcapsules (Figure 1a).

To test the effect of *S*. *spongiosa* polysaccharides on the growth of mice with colitis, the initial and final weights of the mice were recorded. The findings showed that DSS-administered mice suffered severe weight loss compared with the Norm group. Moreover, high *S*. *spongiosa* polysaccharide-based microcapsule intake slightly attenuated weight loss in mice with colitis (Figure 1b).

### 3.2. S. spongiosa Polysaccharides Reduced Disruption of Intestinal Barrier Integrity in Colitis Mice

**Figure 2:**
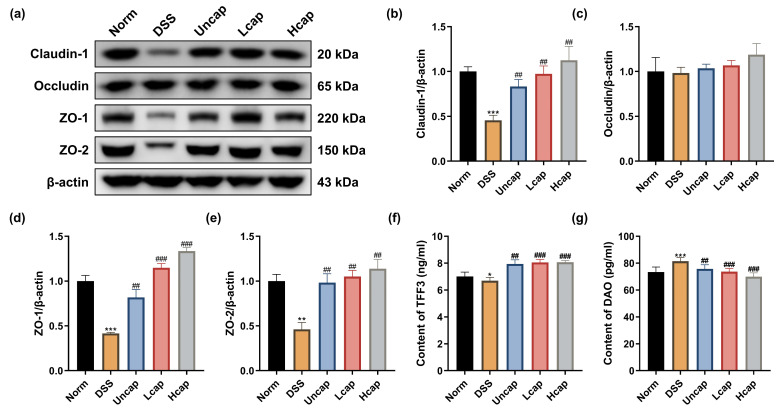
*S. spongiosa* polysaccharides reduced disruption of intestinal barrier integrity in colitis mice. (**a**) The protein abundances of Claudin-1, Occludin, ZO-1 and ZO-2 were estimated by Western blotting analysis. (**b**) The normalization of Claudin-1 to β-actin. (**c**) The normalization of Occludin to β-actin. (**d**) The normalization of ZO-1 to β-actin. (**e**) The normalization of ZO-2 to β-actin. (**f**) The concentration of TFF3 was measured by ELISA. (**g**) The concentration of DAO was measured by ELISA. The values are reported as means ± SD (*n* = 4). * *p* < 0.05, ** *p* < 0.01 and *** *p* < 0.001 vs. Norm group; ^##^ *p* < 0.01 and ^###^ *p* < 0.001 vs. DSS group.

Maintaining the integrity of the intestinal barrier is crucial for the absorption of nutrients and protection against pathogenic microorganism invasion. Hence, we detected the effect of *S*. *spongiosa* polysaccharides on the levels of some tight junction (TJs) proteins. As shown in Figure 2a–e, DSS-induced intestinal barrier disruption resulted from reduced Claudin-1, ZO-1 and ZO-2 activity. *S*. *spongiosa* polysaccharide supplementation alleviated the damage of tight junction proteins induced by DSS. Furthermore, we noticed that the treatment of polysaccharides in the form of microcapsules revealed better protection than that in the Uncap group. We also tested two enzymes trefoil factor 3 (TTF3), which can protect gastrointestinal mucosa and diamine oxidase (DAO), a marker which reflects the degree of damage to the function of the intestinal mucosal barrier. Both uncapped polysaccharides and microcapsules elevated the content of TTF3 (Figure 2f) and diminished the content of DAO (Figure 2g), which suggested that *S*. *spongiosa* polysaccharides benefit intestinal health by reducing disruption of intestinal barrier integrity in mice with colitis.

### 3.3. S. spongiosa Polysaccharides Improved Antioxidant Capacity in Colitis Mice

**Figure 3:**
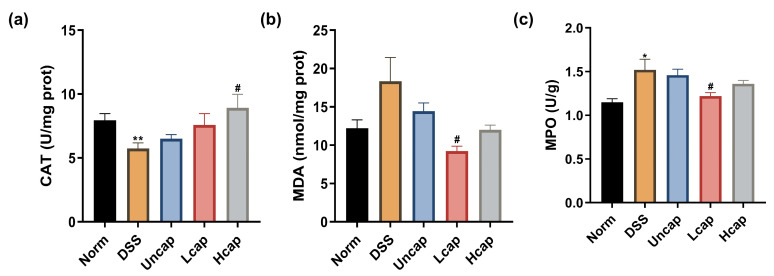
*S. spongiosa* polysaccharides improved antioxidant capacity in colitis mice. The concentrations of CAT (**a**), MDA (**b**) and MPO (**c**) were estimated by ELISA. The values are reported as means ± SD (*n* = 8). * *p* < 0.05 and ** *p* < 0.01 vs. Norm group; ^#^ *p* < 0.05 vs. DSS group.

It is believed that oxidative stress is associated with a variety of intestinal disorders, including UC. The MDA and MPO level and CAT activity in vivo are important indices to evaluate the oxidative stress level of organisms. In the present study, we found that DSS administration disrupted the host antioxidative capacities as decreased CAT content and increased MDA and MPO contents. However, the level of oxidative stress was suppressed by *S. spongiosa* polysaccharides (Figure 3). We also found that the microcapsule technology promoted its antioxidative activity compared with the Uncap group.

### 3.4. S. spongiosa Polysaccharides Alleviated Inflammatory Response in Colitis Mice

**Figure 4:**
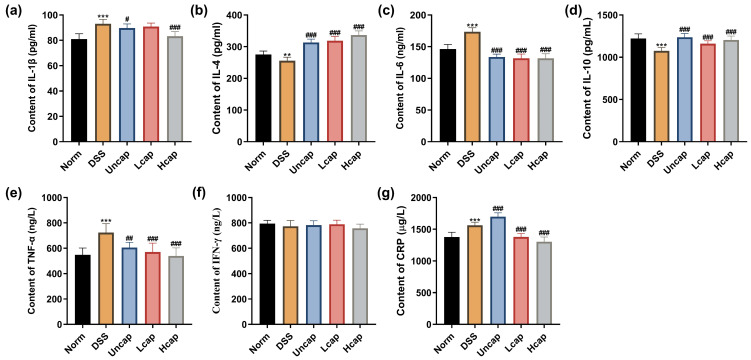
*S. spongiosa* polysaccharides alleviated inflammatory response in colitis mice. The concentration of IL-1β (**a**), IL-4 (**b**), IL-6 (**c**), IL-10 (**d**), TNF-α (**e**), IFN-γ (**f**) and CRP (**g**) were estimated by ELISA. The values are reported as means ± SD (*n* = 8). ** *p* < 0.01 and *** *p* < 0.001 vs. Norm group; ^#^ *p* < 0.05, ^##^ *p* < 0.01 and ^###^ *p* < 0.001 vs. DSS group.

To determine the beneficial role of *S*. *spongiosa* polysaccharides on DSS-triggered inflammation in mice, we assessed serum cytokines using ELISA kits. The *S*. *spongiosa* polysaccharide-based microcapsules significantly decreased the contents of pro-inflammatory cytokines (IL-1β, IL-6, TNF-α and CPR) and increased the contents of anti-inflammatory cytokines (IL-4 and IL-10) in a dose-dependent manner, which was better than the uncapped *S*. *spongiosa* polysaccharides (Figure 4).

### 3.5. S. spongiosa Polysaccharides Modulate the NF-κB Signaling in Colitis Mice

**Figure 5:**
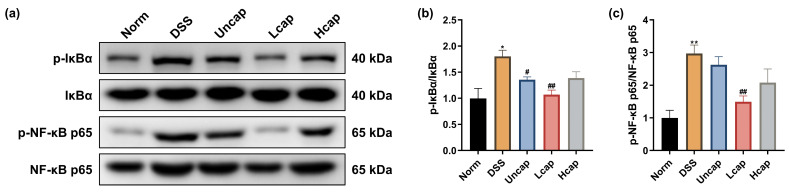
*S. spongiosa* polysaccharides modulate the NF-κB pathway in colitis mice. (**a**) The protein abundances of p-IκBα, IκBα, p-NF-κB p65 and NF-κB p65 were estimated by Western blotting analysis. (**b**) The normalization of p-IκBα to IκBα. (**c**) The normalization of p-NF-κB p65 to NF-κB p65. The values are reported as means ± SD (*n* = 8). * *p* < 0.05 and ** *p* < 0.01 vs. Norm group; ^#^ *p* < 0.05 and ^##^ *p* < 0.01 vs. DSS group.

To uncover the possible mechanisms of *S. spongiosa* polysaccharides in regulating the inflammatory response, we first determined the effect of polysaccharides on the NF-κB signaling pathway in DSS-challenged mice. From the results in Figure 4, DSS administration induced aberrant phosphorylation of IκBα and NF-κB p65. However, *S. spongiosa* polysaccharides decreased the abundances of IκBα and NF-κB p65, in which Lcap polysaccharides exerted better protective activity (Figure 5).

### 3.6. S. spongiosa Polysaccharides Modulate the JAK/STAT Signaling in Colitis Mice

**Figure 6:**
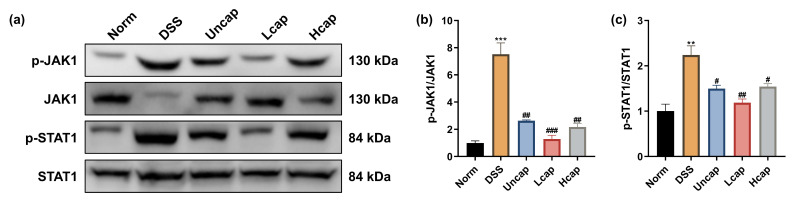
*S. spongiosa* polysaccharides modulate the JAK/STAT pathway in colitis mice. (**a**) The protein abundances of p-JAK1, JAK1, p-STAT1 and STAT1 were estimated by Western blotting analysis. (**b**) The normalization of p-JAK1 to JAK1. (**c**) The normalization of p-STAT1 to STAT1. The values are reported as means ± SD (*n* = 8). ** *p* < 0.01 and *** *p* < 0.001 vs. Norm group; ^#^ *p* < 0.05, ^##^ *p* < 0.01 and ^###^ *p* < 0.001 vs. DSS group.

Furthermore, we detected the JAK/STAT signaling cascade. DSS administration reinforced the phosphorylation states of JAK1 and STAT1. In contrast, both uncapped and microcapsules decreased the protein abundances of p-JAK1 and p-STAT1 (Figure 6), implying that *S*. *spongiosa* polysaccharides harbor the potential for mitigating inflammatory response which is associated with impeding the activation of the JAK-STAT signaling cascade.

### 3.7. S. spongiosa Polysaccharides Modulate the MAPK/ERK Pathway in Colitis Mice

**Figure 7:**
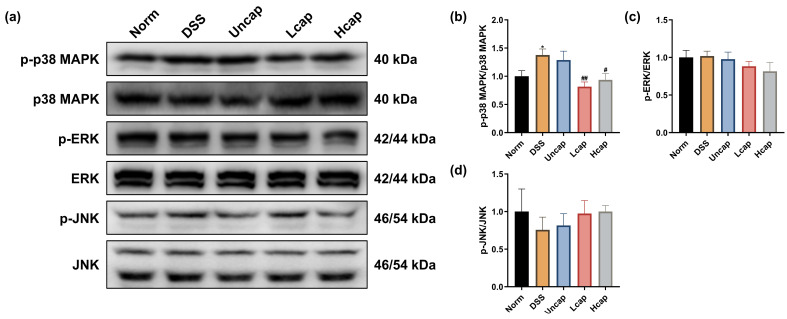
*S. spongiosa* polysaccharides modulate the MAPK/ERK pathway in colitis mice. (**a**) The protein abundances of p-p38 MAPK, p38 MAPK, p-ERK, ERK, p-JNK and JNK were estimated by Western blotting analysis. (**b**) The normalization of p-p38 MAPK to p38 MAPK. (**c**) The normalization of p-ERK to ERK. (**d**) The normalization of p-JNK to JNK. The values are reported as means ± SD (*n* = 8). * *p* < 0.05 vs. Norm group; ^#^ *p* < 0.05 and ^##^ *p* < 0.01 vs. DSS group.

Multiple stimuli-triggered activations of the MAPK/ERK pathway have been demonstrated to be associated with inflammation. DSS administration was forceful in promoting the protein abundance of p-p38 MAPK. Microcapsule treatment reduced the phosphorylation of p38 MAPK, not ERK or JNK (Figure 7).

### 3.8. S. spongiosa Polysaccharides Remold the Richness and Diversity of Intestinal Microbiota

**Figure 8:**
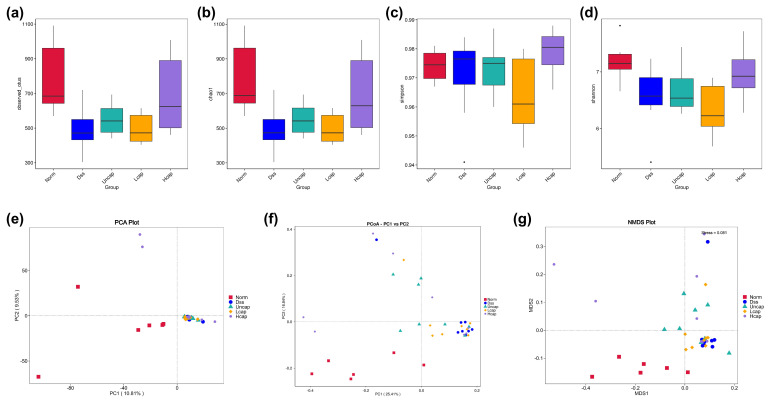
*S. spongiosa* polysaccharides remold the richness and diversity of intestinal microbiota. The alpha diversity including observed OTUs (**a**), Chao (**b**), Simpson (**c**) and Shannon (**d**) indices were measured by 16S rDNA. (**e**) PCA analysis based on ASV level. PCoA (**f**) and NMDS (**g**) analysis based on unweighted UniFrac distances.

A diversity analysis with one single sample size (Alpha diversity) reflects the abundance and diversity of the microbial community. In this study, we noticed that DSS administration disrupted the total OTUs of intestinal microbiota and high-capped *S*. *spongiosa* polysaccharides enhanced the observed OTUs in comparison with the DSS group (Figure 8a). Besides, the Chao1 and Shannon indices were significantly decreased in DSS-challenged mice while returned to normal levels by *S*. *spongiosa* polysaccharides (Figure 8b–d).

Beta Diversity is a comparative analysis of the microbiota composition to find the differences between different samples (groups) through principal component analysis (PCA), principal co-ordinates analysis (PCoA) and non-metric multi-dimensional scaling (NMDS). The results demonstrated that the gut microbial community was altered by either DSS administration or *S*. *spongiosa* polysaccharide treatment, as evidenced by the five groups located in different branches (Figure 8e–g).

### 3.9. S. spongiosa Polysaccharides Remold the Composition of Intestinal Microbiota

**Figure 9:**
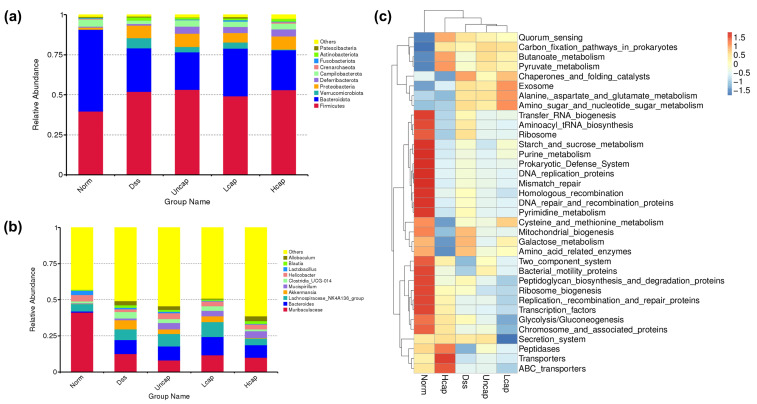
*S. spongiosa* polysaccharides remold the composition of intestinal microbiota. (**a**) The microbiota composition and proportion among five groups at the phylum level. (**b**) The microbiota composition and proportion among five groups at the genus level. (**c**) The cluster heatmap of the Tax4Fun Prediction Function among five groups.

At different taxonomic levels, the microbiota composition and proportion of each sample were determined. At the phylum level, DSS administration caused an obvious decrease in the abundance of *Bacteroidota* and *Campilobacterota* and an increase in the abundance of *Firmicutes*, *Proteobacteria* and *Verrucomicrobiota*. Whereas *S*. *spongiosa* polysaccharide-based microcapsules prevented the increase in *Verrucomicrobiota* and enhanced the abundance of *Deferribacterota* and *Actinobacteriota* (Figure 9a). At the genus level, the abundances of *Muribaculaceae*, *Helicobacter* and *Lactobacillus* declined and the abundances of *Bacteroides*, *Lachnospiraceae_NK4A136_group*, Akkermansia and *Clostridia_UCG-014* increased by DSS administration. Partly, high-capped polysaccharides reduced the *Akkermansia* and *Clostridia_UCG-014* abundances and promoted the *Mucispirillum* and *Helicobacter* abundances compared with the DSS group (Figure 9b). Furthermore, Tax4Fun Prediction Function analysis indicated that high-capped polysaccharide treatment facilitated short-chain fatty acid (SCFAs) metabolism and low-capped polysaccharide treatment facilitated amino acid metabolism compared with the DSS group (Figure 9c).

## 4. Discussion

As an increasingly prevalent bowel disease, UC is seldom neglected in its early period, and it is routinely regarded that the pathogenesis is rigorously related to inflammatory processes [29,30]. The inflammatory process of UC is associated with changes in the integrity of the intestinal epithelial barrier and the gut microbial composition [31]. Our investigations suggested that the anti-inflammatory properties of *S*. *spongiosa* polysaccharide and its ability to maintain the intestinal mucosal barrier function as well as ameliorate the intestinal microbiota-associated changes may offer potential clinical therapeutic advantages for relieving the severity of UC. Therefore, our study suggests that *S*. *spongiosa* polysaccharide may be an effective potential therapy for thwarting the processing of UC.

The main functions of the colon are the absorption of water and electrolytes, and the formation, storage and excretion of feces [32]. Typical symptoms such as diarrhea, constipation and abdominal distension occur if the colonic function is disturbed [33]. Therefore, weight loss is often used to assess clinical conditions. Although our results indicated that the DSS-induced weight reduction was not subverted by *S*. *spongiosa* polysaccharide treatment, we found that high-capped *S*. *spongiosa* polysaccharides mildly attenuated weight loss in mice with colitis.

Oxidative stress refers to an imbalance of oxidative and antioxidant effects in the body, leading to the production of large quantities of oxidative intermediates which are implicated in the pathogenesis of colitis and related carcinogenesis [34,35]. Reactive oxygen species (ROS) play an important role in the occurrence and development of UC, not only promoting lipid peroxidation to generate MDA but also inducing inflammatory infiltration of neutrophils on colon tissue [36]. CAT, one of the antioxidant enzymes, is the first line of defense against oxidative stress, protecting cells from oxidative damage by scavenging oxygen free radicals and lipid peroxides [37]. Multiple polysaccharides obtained from natural sources have been considered as a potential therapy for intestinal injury due to their function to relieve oxidative stress [38]. Here, we demonstrated that the concentrations of MDA and MPO increased and CAT decreased in mice with colitis. *S*. *spongiosa* polysaccharide-based microcapsules alleviated the oxidative stress induced by DSS, implying that *S*. *spongiosa* polysaccharides could alleviate oxidative stress damage induced by DSS to some extent.

Inflammatory mediators (cytokines and chemokines, etc.) have an important relationship with IBD, and their excessive production can exacerbate the inflammatory cascade, further causing colon damage [39,40]. Increasing evidence suggested that intestinal injury is associated with a complex interplay between immune and non-immune cells that leads to local inflammation [41]. Overactivation of the intestinal immune response is thought to be one of the important drivers of inflammation and related disease progression, and the number of immune cells correlates with increased production of inflammatory cytokines. Among them, pro-inflammatory cytokines play an important role in the recruitment of immune cells. IL-6, a key mediator of inflammation in UC, can activate the STAT3 and cause the accumulation of CD4^+^T lymphocytes, resulting in chronic inflammation [42,43]. The upregulated IL-1β levels may be related to the recruitment and retention of leukocytes from patients with inflammatory diseases and the stimulation of the innate immune T lymphocytes (IHLs) [44]. In IBD, IL-4 is produced by Th2-type CD4^+^T cells and can counteract the generation of Th1-type inflammatory factors such as IL-2 and IFN-γ [45]. Therefore, preventing the secretion of pro-inflammatory cytokines in the intestinal microenvironment may be a feasible therapeutic approach [46]. Our study proved that *S*. *spongiosa* polysaccharides dose dependently decreased the contents of pro-inflammatory cytokines (IL-1β, IL-6, TNF-α and CPR) and increased the contents of anti-inflammatory cytokines (IL-4, and IL-10), in which high-capped microcapsules showed a stronger effect. These results suggest that *S*. *spongiosa* polysaccharides partially alleviate the pathogenesis of UC by settling the balance of inflammatory responses. Considerable evidence points to a role for NF-κB, MAPK and JAK/STAT cascades in the development of UC by regulating multiple inflammatory cytokines’ production [47,48,49]. Hence, we detect the expression of key proteins associated with these signaling pathways. Here, we found that the phosphorylation of IκBα, NF-κB p65, JAK1, STAT1 and p38 MAPK was promoted in DSS-challenged mice and reduced by *S*. *spongiosa* polysaccharide treatment. Briefly, our study demonstrated that *S*. *spongiosa* polysaccharides maintain the inflammatory response in DSS-triggered colitis mice through regulating the activation of NF-κB/STAT/MAPK signaling.

Changes in TJ protein expression/cell distribution have been shown to affect mucosal inflammation by regulating mucosal homeostasis and intestinal permeability [50,51]. As the most used UC modelling approach, the mechanism by which DSS causes colonic inflammation and barrier damage remains unclear. The structure and location of TJs in the colon are disrupted, allowing bacterial penetration and inflammation induction [52]. Furthermore, the expression of TJs like Claudin, Occludin and ZO-1 proteins may be reduced by pro-inflammatory factors like IL-6 and TNF-α, which impair the barrier ability of the intestine [53,54]. Occludin, Claudin-1 and ZO-1 are intestinal epithelial tight junction marker proteins, and ZO-1 and Occludin were considered as valuable predictive proteins for the severity of colitis and mucosal healing. In the present investigation, we found that *S. spongiosa* polysaccharide supplementation restored the damage of tight junction proteins (Claudin-1, Occludin, ZO-1 and ZO-2) induced by DSS. Additionally, we evaluated the contents of DAO and TFF3, markers of intestinal integrity. Coinciding with the results of TJs, *S. spongiosa* polysaccharides improved intestinal barrier integrity by enhancing TFF3 concentration and reducing DAO activity. Although more work is needed to clarify the underlying mechanisms of their action, this study tentatively suggests that *S. spongiosa* polysaccharides can restore the integrity of the intestinal epithelial barrier in a mouse model of UC induced by DSS, thereby alleviating the effects of UC.

The gut flora (GP) is important in gut health, directly associated with the integrity of the intestinal epithelial barrier [55,56]. A large number of bioactive molecules such as peptide elastase inhibitors isolated from natural plants have been proven as a potential target for the prevention of chronic diseases by regulating intestinal microbiota changes in COVID-19 patients [57,58]. There are a variety of chronic inflammatory conditions which may be caused or aggravated by an imbalance in gut flora, including UC [59]. In contrast to healthy individuals, UC patients exhibit a unique pattern of intestinal microbial diversity and abundance. Numerous studies have demonstrated that multiple bioactive nutrients derived from foods influence the composition and diversity of gut microbiota through direct interactions or mediating signaling pathways [60,61,62]. In this study, the richness in microcapsule-administered mice was higher than that in the DSS group, as shown in increased observed OTUs, Shannon and Chao1 indices. In addition, the composition of *Firmicutes*, *Bacteroidota*, *Deferribacterota* and *Actinobacteriota* was altered by *S. spongiosa* polysaccharides, suggesting that these microorganisms may play a critical role in the prevention of colonic lesions induced by DSS.

## 5. Conclusions

In summary, this study substantiated that *S. spongiosa* polysaccharides exhibited protective effects against colitis mice by reshaping the intestinal microbiome and maintaining the balance of intestinal barrier integrity, oxidative capacity and colonic inflammation through the regulation of NF-κB–STAT1–MAPK axis. Thus, our findings provide a novel insight that *S. spongiosa* polysaccharides may serve as a therapeutic strategy for the treatment of IBD.

## Data Availability

All data is available within the article.
